# Prevalence and risk factors for Subclinical Rheumatic Heart Disease among primary school children in Dar es Salaam, Tanzania: a community based cross-sectional study

**DOI:** 10.1186/s12872-021-02377-9

**Published:** 2021-12-20

**Authors:** Parvina Titus Kazahura, Theophylly L. Mushi, Pedro Pallangyo, Mohamed Janabi, Rodrick Kisenge, Mazen Albaghdadi, Naizihijwa Majani, Edward Kija

**Affiliations:** 1 Department of Paediatrics and Child Health, Muhimbili University of Health and Allied Sciences, P.O Box 65001, Dar es Salaam, Tanzania; 2 Department of Paediatric Cardiology, Jakaya Kikwete Cardiac Institute, P.O Box 54141, Dar es Salaam, Tanzania; 3 Department of Adult Cardiology, Jakaya Kikwete Cardiac Institute, P.O Box 54141, Dar es Salaam, Tanzania; 4 Division of Cardiology and Section of Vascular Medicine, Massachusetts General Hospital, Harvard Medical School, Boston, MA USA

**Keywords:** Subclinical RHD, Valvular heart lesions, Echocardiography screening, Rheumatic Fever, Tanzania

## Abstract

**Background:**

Rheumatic heart disease (RHD) is the most common acquired heart disease occurring in children and adolescents. RHD is associated with significant morbidity and mortality particularly in low and middle- income countries (LMICs) where the burden is estimated to be higher compared to high income countries. Subclinical RHD is the presence of valvular lesion diagnosed by echocardiography in a person with no clinical manifestation of RHD. This study aimed at determining the prevalence, types and factors associated with subclinical RHD among primary school children in Dar Es Salaam, Tanzania.

**Methods:**

A descriptive community-based cross-sectional study was conducted in primary school children from February to May 2019. A standardized structured questionnaire was used to collect demographic characteristics, history of upper respiratory tract infections (URTIs), anthropometric measurements, and chest auscultation findings. Moreover echocardiographic screening was done to all children recruited into the study. World Heart Federation echocardiographic classification was used to define the types and prevalence of subclinical RHD.

**Results:**

A total of 949 primary school children were enrolled with females being predominant (57.1%). The prevalence of subclinical RHD was 34 per 1000. All the participants had mitral valve disease only whereby 17 had definite disease and 15 had a borderline disease. The associated factors for subclinical RHD were older age of more than 9 years (OR 10.8, 95% CI 1.4–82.2, *P* = 0.02) having three or more episodes of URTI in previous six months (OR 21, 95% CI 9.6–46, *P* = 0.00) and poor hygiene (OR 3, 95% CI 1.3–6.8, *P* = 0.009).

**Conclusion:**

Subclinical RHD as detected by echocardiographic screening is prevalent in primary school children, uniformly affects the mitral valve, and is associated with potentially modifiable risk factors. Children with a history of more than three episodes of URTI in six months represents a high-risk population that should be targeted for RHD screening.

## Background

Rheumatic fever (RF) is a multi-system, post infectious inflammatory disease, which presents as a delayed sequela to Group A streptococcal (GAS) pharyngitis. In developing countries it remains a major health concern due to poor health seeking behaviour, overcrowding, poor nutrition and scarcity of health care resources [1]. RF is mainly a disease of children aged 5 to 14 years old and rare in persons above 30 years [2]. It is hypothesized to be due to an immune mediated pathogenesis secondary to GAS infection.

Rheumatic heart disease (RHD) is the most serious complication of rheumatic fever whereby patients develop heart valve regurgitation or stenosis, atrial dilation, arrhythmias and right ventricular dysfunction [3]. After a patient has had RF, there is often a prolonged period of subclinical disease characterized by changes in valvular morphology and function [4]. It has been shown that 40 to 65% of patients who have had RF get clinically recognizable RHD [5, 6]. Subclinical RHD is a term used to describe the presence of morphological and functional valvular lesions detected by echocardiography but with no corresponding heart murmur [7]. The latent period of subclinical RHD provides a window of opportunity for screening, initiation of secondary prophylaxis, and referral for valvular intervention when appropriate.

At the Jakaya Kikwete Cardiac Institute (JKCI) in Dar es Salaam, Tanzania, 29.5% and 32.9% of all cardiac surgeries in 2017 and 2018 respectively were performed to treat RHD “(unpublished data)”. The World Heart Federation continues to recommend screening as a component of RHD control in high prevalence areas [8]. However, there is a paucity of data in Sub-Saharan Africa with regard to RHD and associated risk factors, particularly in children [9, 10]. In the current study, we utilized WHF echocardiographic screening criteria and a structured questionnaire to evaluate the prevalence and risk factors of subclinical RHD, respectively, in Tanzanian primary school children.

## Methods

A community based descriptive cross-sectional study was conducted at Muhimbili and Mjimpya primary schools in Dar es salaam, Tanzania. Both are public, mixed-sex schools, and the former represents a middle-class population while the latter represents a lower socioeconomic class population. Muhimbili primary school had a total number of 1120 students while Mjimpya primary school had a total number of 1067 students.

School children aged 7–18 years were recruited from February to May 2019. All students from grade 1 to grade 7 were provided with questionnaires and consent forms for parents to fill at home and children were asked for assent. Those who brought back the filled questionnaires with a signed consent within two weeks after being given were consecutively recruited for the study.

All the children with established rheumatic heart disease determined by transthoracic echocardiography prior to the conduction of this study,and those who denied consent/assent were excluded from the study.

The risk factors, socioeconomic and demographic characteristics, hygiene status, overcrowding, and history of sore throat in the previous six months were recorded. Those who had at least 3 episodes of upper respiratory tract infectious symptoms were regarded to have recurrent upper respiratory tract infection (URTI). Anthropometric measurements for height, weight and BMI were interpreted from WHO BMI charts for children aged 5–19 years.

Cardiac auscultation and echocardiographic screening were conducted at school in a room with a door and curtains. Cardiac auscultation using a stethoscope and transthoracic Doppler echocardiography (2D echo) (Siemens ACUSON P500) with a paediatric cardiac probe were performed by P.K and T.L.M. 2D echo images were taken using the apical four chamber view, parasternal long and short axes views, with and without colour Doppler. Mitral and aortic valve leaflets, mitral valve chordae, regurgitant jets, valve coaptation and flow velocity across the valves were viewed and recorded. Screened participants found to have any morphological valvular pathology as per World Heart Federation (WHF) criteria were referred to JKCI for detailed echocardiography that was performed by the paediatric cardiologist using a Siemens ACUSON X300 PE Premium Edition. The interpretations of echocardiographic findings were defined according to the WHF echocardiographic criteria for RHD as shown in Table 1 [11].

**Table 1:** WHF criteria for echocardiographic diagnosis of rhd for individuals ≤ 20 years

Definite RHD (either A, B or C)
(A) Pathological MR and at least two morphological features of RHD of the MV
(B) MS mean gradient ≥ 4 mmHg
(C) Pathological AR and at least two morphological features of RHD of the AV
(D) Borderline disease of both the AV and MV
Borderline RHD (either A, B or C)
(A) At least two morphological features of RHD of the MV without pathological MR or MS
(B) Pathological MR
(C) Pathological AR
Normal echocardiographic findings (all of A, B, C and D)
(A) MR that does not meet all four Doppler echocardiographic criteria (physiological MR)
(B) AR that does not meet all four Doppler echocardiographic criteria (physiological AR)
(C) An isolated morphological feature of RHD of the MV (for example, vulvular thickening) without any associated pathological stenosis or regurgitation
(D) Morphological feature of RHD of the AV (for example, vulvular thickening) without any associated pathological stenosis or regurgitation
Normal echocardiographic findings (all of A, B, C and D)
(A) MR that does not meet all four Doppler echocardiographic criteria (physiological MR)
(B) AR that does not meet all four Doppler echocardiographic criteria (physiological AR)
(C) An isolated morphological feature of RHD of the MV (for example, vulvular thickening) without any associated pathological stenosis or regurgitation
(D) Morphological feature of RHD of the AV (for example, vulvular thickening) without any associated pathological stenosis or regurgitation
Criteria for Pathological mitral regurgitation
(All four Doppler echocardiographic criteria must be met)
Seen in two views (apical four chamber view and parasternal long axis or short axis view)
In at least one view, jet length ≥ 2 cm
Velocity ≥ 3 m/s for one complete envelope
Pan-systolic jet in at least one envelope
Criteria for Pathological aortic regurgitation
(All four Doppler echocardiographic criteria must be met)
Seen in two views
In at least one view, jet length ≥ 1 cm
Velocity ≥ 3 m/s in early diastole
Pan-diastolic jet in at least one envelope
Morphological features of RHD in the MV
AMVL thickening ≥ 3 mm
Chordal thickening
Restricted leaflet motion
Excessive leaflet tip motion during systole
Morphological features of RHD in the AV
Irregular or focal thickening
Coaptation defect
Restricted leaflet motion
Prolapse

*WHF* World Heart Federation, *MR* mitral regurgitation, *RHD* rheumatic heart disease, *MV* Mitral valve, *MS* mitral stenosis, *AR* Aortic regurgitation, *AV* aortic valve, *AMVL* anterior mitral valve leaflet

Statistical analysis was done using SPSS version 20.0 (IBM®), statistical software for data analysis. Continuous variables were summarized presented as means with standard deviations. Categorical variables were presented as proportions. A univariate regression analysis using Chi-square was performed to examine predictors of subclinical RHD. All factors with a *P* value < 0.2 were included in the logistic regression model. All statistical analyses were two sided and a *P* value < 0.05 was used to denote statistical significance.

## Results

**Fig. 1:**
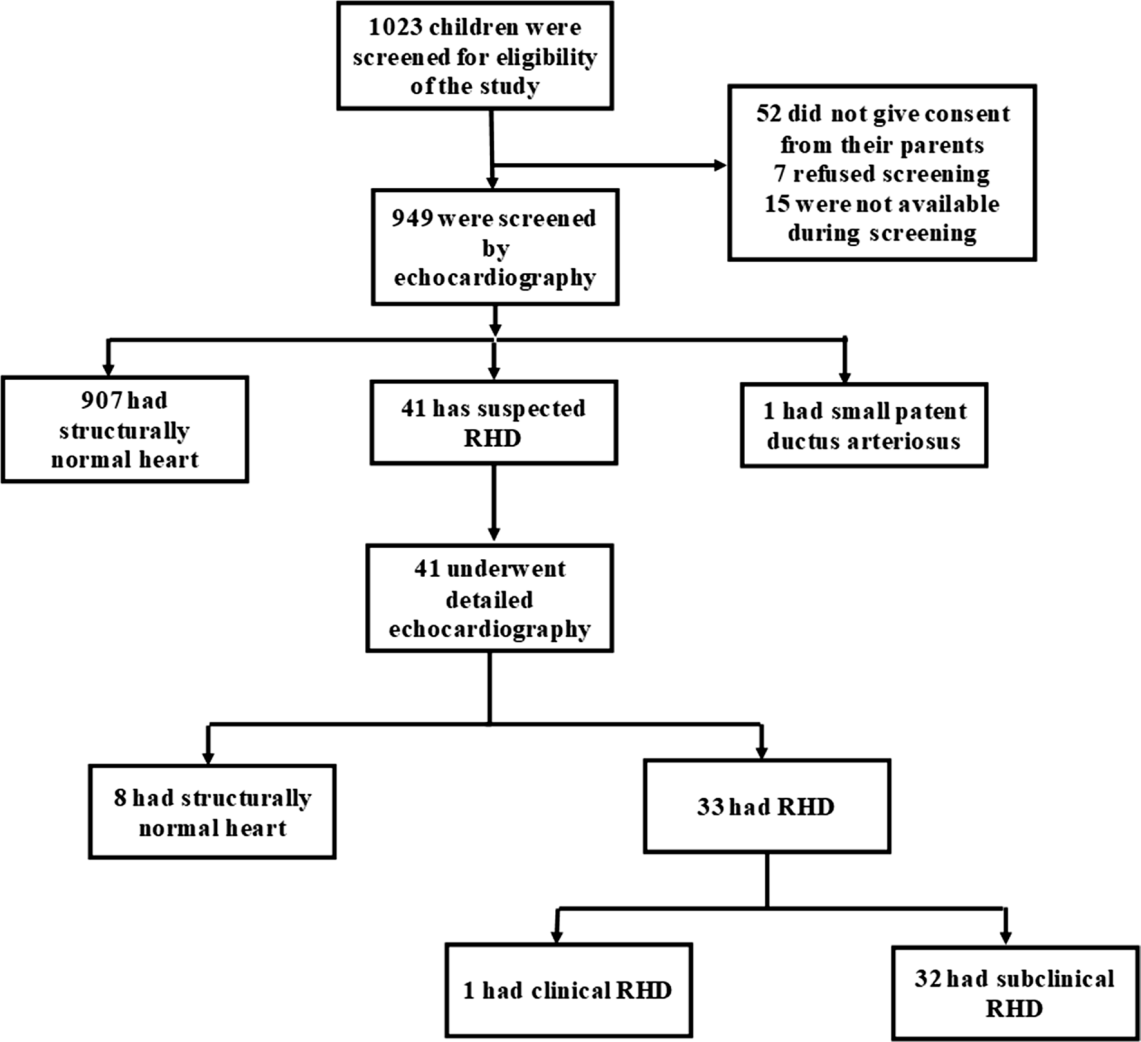
CONSORT flow diagram

A total of 1023 children were screened for eligibility and 949 were enrolled into this study (Fig. 1). Table 2 displays the socio-demographic characteristics of the study population. The mean age of participants was 10.8 ± 1.7 and their ages ranged from 7 to 17 years. There was a female predominance (57.1%) and over three quarters of all participants had a normal BMI. Most parents were small scale business owners and had attained primary school as their highest level of education.

**Table 2:** Socio-demographic characteristics of the study participants (n = 949)

Demographics	Mean ± SD/frequency (%)	
Age of children, Mean ± SD; Range	10.8 ± 1.7, 7–17 years	
Sex N (%); Female, Male	542 (57.1); 407 (42.9)	
BMI N (%)		
Normal	742 (78.2)	
Severe underweight	20 (2.1)	
Underweight	156 (16.4)	
Overweight	23 (2.4)	
Obese	8 (0.8)	
Age of the parents	Mother	Father
Mean ± SD	36 ± 6.6	42 ± 9.3
Range	22–54 years	23–76 years
Parents’ education level N (%)	Mother	Father
No formal education	28 (3)	12 (1.3)
Primary education	461 (48.6)	327 (34.5)
Secondary education	298(31.4)	336 (35.4)
Above secondary education	140 (14.8)	198 (20.9)
Parents’ occupation N (%)	Mother	Father
None	224 (23.6)	37 (3.9)
Rural Farmer	23 (2.4)	50 (5.3)
Small scale business	517 (54.5)	417 (43.9)
Large scale business	43 (4.5)	114 (12)
Employed	127 (13.4)	264 (27.8)
People living in one house N (%)	> 6 people	< 6people
	315 (33.2)	595 (62.7)
People sharing bedroom with the child N (%)	> 2	< 2
	206 (21.7)	692 (72.9)

About one third of all participants came from families of six or more people. The screened child shared a bedroom with at least two other family members in over 21% of families.

### Prevalence of subclinical RHD among primary school children

**Fig. 2:**
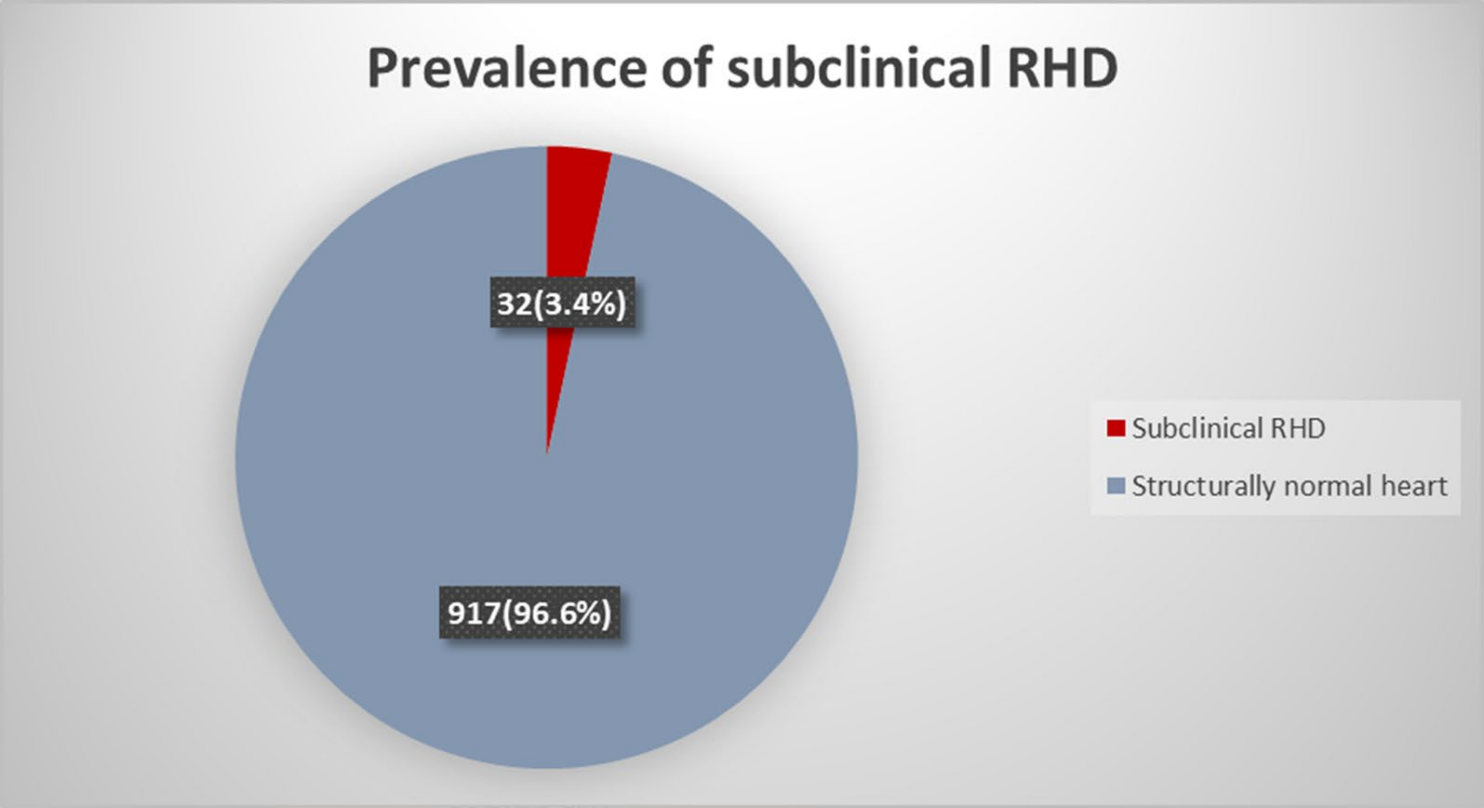
Prevalence of subclinical RHD among screened primary school children in Dar es salaam. (n = 949)

As shown in Fig. 2, a total of 949 asymptomatic primary school children were recruited and screened for subclinical RHD. Thirty two (23 girls and 9 boys) were found to have subclinical RHD making a prevalence of 34 per 1000, out of which 17 had a definite disease and 15 had a borderline disease. The prevalence of subclinical RHD did not differ between Muhimbili and Mjimpya primary schools (3.2% and 3.6% respectively). One child had clinically detected RHD by a grade 3 pan systolic murmur on the apex, which was later confirmed by echocardiography with the presence of pathological mitral regurgitation (MR) and anterior mitral valve leaflet (AMVL) thickening. Also one child was diagnosed with a 2 mm patent ductus arteriosus with a pressure gradient of 65 mmHg.

### Types of valvular lesions

**Fig. 3:**
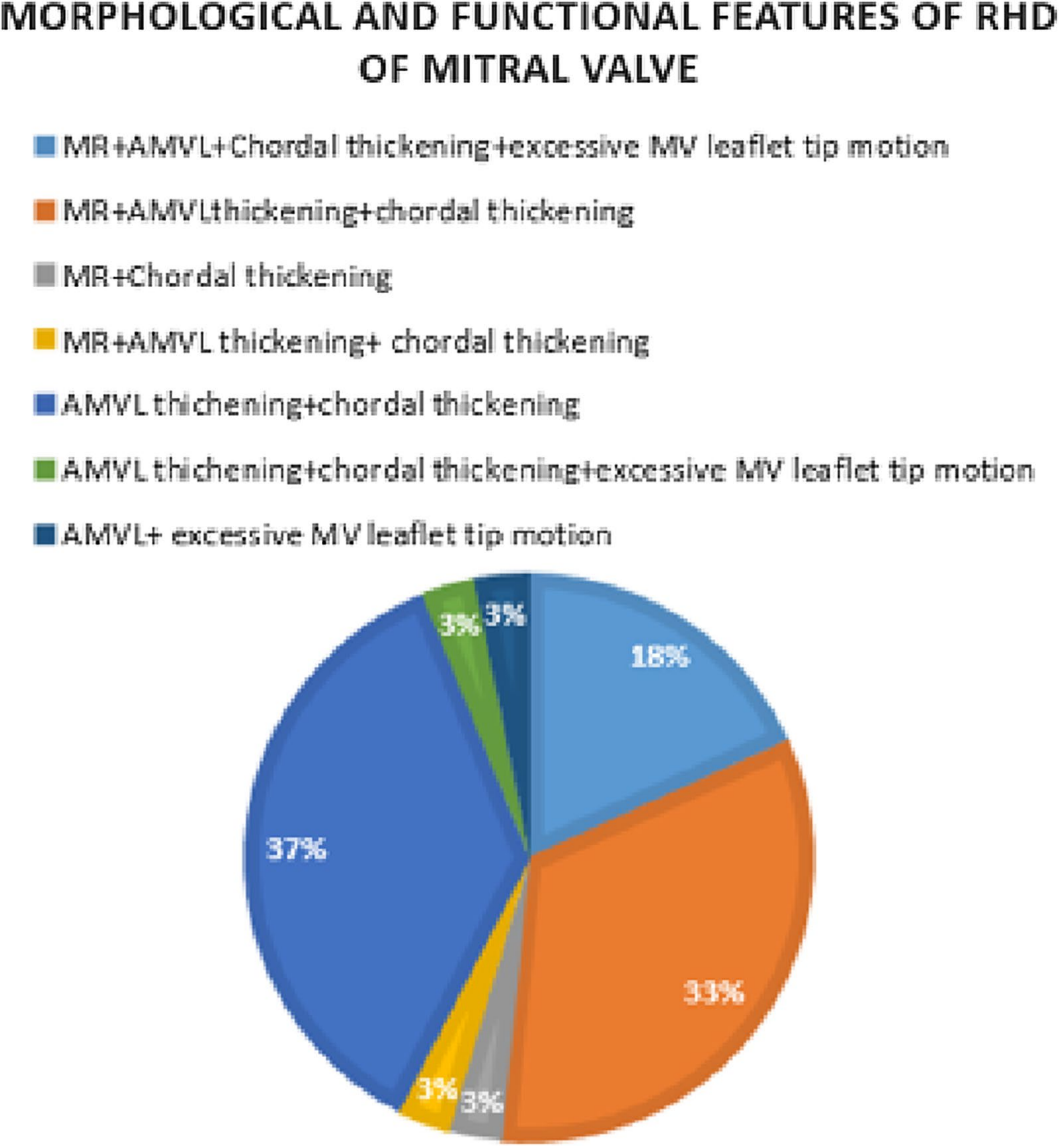
Morphological and functional features of RHD of the MV. The prevalence of various (n = 33)

All 32 primary school children who were found to have subclinical RHD had lesions of the mitral valve. There was no child with mitral stenosis or disease of the aortic valve as shown in Fig. 3. The following valvular lesions were most commonly observed: anterior mitral valve leaflet (AMVL) thickening > 3 mm and chordal thickening in 12 out of 32 (36.5%), and pathological MR in 11 out of 32 (33%).

### Socioeconomic and clinical risk factors for subclinical RHD

Risk factors that were associated with subclinical RHD are shown in Table 3. Recurrent URTI, poor hygiene and age greater than nine years were associated with the risk of subclinical RHD on univariate analysis. Multivariate logistic regression model showed, as shown in Table 4, School children aged more than 9 years, recurrent URTI and poor hygiene showed statistical significance for subclinical RHD.

**Table 3:** Univariate predictors of subclinical RHD

Factor	N (%)	Chi-square	*p* value
Age			
> 9 years	31 (4.3)	8.3	0.004
* ≤ 9 years	1 (0.4)		
Hygiene			
Poor	22 (4.9)	5.9	0.015
*Good	10 (2)		
Overcrowding			
Yes	19 (4.6)	3	0.08
*No	13 (2.5)		
Mothers education			
Low	18 (3.9)	0.16	0.69
*High	14 (3.2)		
Family income			
Low	20 (4)		
*Middle	12 (2.9)	1.7	0.19
URTI			
Recurrent	20 (22)	29.3	0.00
*Non-recurrent	12 (4.1)		
Nutrition			
Underweight	8 (4.5)	0.9	0.34
*Not underweight	24 (3.1)		

*Reference

**Table 4:** Multivariate predictors of subclinical RHD

Factor	COR (95% CI)	*p* value	AOR (95% CI)	*p* value
Age				
> 9 years	10.6 (1.4–78.2)	0.02	10.8 (1.4–82.2)	0.02
*≤ 9 years				
Hygiene				
Poor	2.5 (1.2–5.3)	0.02	3 (1.3–6.8)	0.009
*Good				
Overcrowding				
Yes	1.9 (0.9–3.8)	0.09	–	-
*No				
Family income				
Low	1.7 (0.8–3.6)	0.19	–	-
*Middle				
URTI				
Recurrent				
*Non-recurrent	19.6 (9.1–41.2)	0.00	21 (9.6–46)	0.00

*Reference

## Discussion

Echocardiographic screening for the identification of subclinical RHD is the gold-standard method for understanding disease burden and severity in endemic regions. Insights from echocardiography-based RHD screening programs are vital to informing advocacy and public health responses to reduce the burden of RHD, however data from sub-Saharan Africa are limited. In this study, we sought to provide insights into the epidemiology and echocardiographic characteristics of subclinical RHD in Tanzanian school-children. We found that subclinical RHD as detected by echocardiographic screening is not uncommon in primary school children in Dar es salaam, uniformly affects the mitral valve, and is associated with potentially modifiable risk factors.

We observed an overall prevalence of subclinical RHD to be 34 per 1000. The prevalence of subclinical RHD in this study is comparable to that found in other studies in Africa including: Senegal, Brazzaville, Uganda, Ethiopia, Mozambique and Malawi which ranges between 4.95 per 1000 and 32.6 per 1000 [12–16]. Furthermore our findings are very similar to the reported prevalence of subclinical RHD in Malawi with the difference being in the number of definite and borderline cases whereby their study had more borderline cases than definite cases compared to this study. The similarity could be explained by both studies having been conducted in sub-Saharan Africa where sociodemographic characteristics are similar [12]. The number of definite cases in this study was slightly higher than the number of borderline cases which was also demonstrated in the studies done in Eastern Nepal and Mozambique. This is due to the fact that in all three studies the RHD was more prevalent in children of 9 years of age and above when significant valvular changes have taken place for a definite disease to occur [13, 17]. The prevalence of RHD detected by clinical examination was approximately 1 per 1000 compared to the prevalence detected by echocardiography which was approximately 34 per 1000. Several studies have shown that, regardless of the experience of the examiner, the sensitivity and specificity of echocardiography is greater than cardiac auscultation [18–20]. The early progressive valvular changes in RHD are silent and hence it is difficult to pick them by cardiac auscultation unless they are visualized by echocardiography.

In this study, all the participants who were found to have RHD had mitral valve disease without involvement of any other valve. Typical features of mitral valve disease observed in our study include regurgitation, AMVL thickening, chordal thickening and excessive mitral valve leaflet motion. Several studies from Sub-Saharan Africa have highlighted the predominance of mitral valve disease among individuals with subclinical RHD. Other studies have shown that aortic valve disease may be associated with mitral valve disease though only in a small percentage of individuals [5, 21]. Pure mitral stenosis is commonly seen in the third decade of life and given that our study population was all less than the age of 17, we did not observ rheumatic mitral stenosis as would be expected in this younger population [22]. This pattern is similar to that seen by Chimalizeni et al.in Malawi school children screened whereby mitral regurgitation was the most common valvular lesion, there was only one child with aortic valve disease and there was no mitral stenosis [12]. Moreover, this finding is in agreement with other RHD echocardiographic screening studies in Cambodia, Mozambique and Senegal which also were conducted in school children to determine the prevalence of subclinical RHD. These studies showed that the mitral valve was the most affected valve followed by aortic valve disease. Mitral stenosis was not observed [13, 14] In subclinical RHD, mitral stenosis is not a common lesion of mitral valve disease as it has not been reported in several studies except the screening done in school children from Ethiopia where mitral stenosis was found in 7% of children [16]. The mitral valve is commonly involved in RHD probably because the mitral valve cusps are exposed to the pressure of the left ventricle during contraction in systole but the aortic cusps are exposed to the aortic diastolic pressure during closure, so the shear stress on the large mitral leaflets is more than on the small aortic cusps thus making the mitral valve more prone to injury during the RF attacks. The findings of the current study have important implications for the design of future echocardiographic screening studies that could be directed at only the mitral valve in resource-limited settings.

Multiple studies from Sub-Saharan Africa, including Mozambique, Uganda, Senegal, and Malawi, have observed that children above the age of 9 years have a higher observed prevalence of subclinical RHD compared to younger children [12–15, 17]. At an older age of 9 years and above, there are notable valvular changes after the child has had a number of RF attacks. Although the development of RHD is associated with poor hygiene [23], few studies have examined the association between hygiene, respiratory tract infections, and RHD. Not adhering to handwashing practices has been associated with predisposing a child to streptococcal infections like impetigo which is regarded as a risk factor for URTI [23]. In a systematic review by Wilson et al.exploring the impact of simple hygiene interventions introduced in primary schools and day care centres on respiratory and gastrointestinal infections, it was found that hand hygiene can reduce the incidence of URTI [24]. In Pakistan, poor hygienic conditions were reported as a major risk factor for RF and RHD, however, their sample size was smaller compared to this study and included only children diagnosed with acute rheumatic fever or RHD in the outpatient clinic by echocardiography [23]. Our findings are also consistent with those reported by Ngaide et al. from Senegal and Vlajinac et al. in Yugoslavia, where repeated sore throat was observed to be a predisposing factor for the development of RHD especially for those who had definite RHD by echocardiography [14, 25]. Similarly, in the present study, we observed that children aged more than 9 years, recurrent URTI, and poor hygiene were associated with prevalent RHD. Our results highlight the importance and relevance of current guidelines and expert opinion regarding the prevention of rheumatic fever and rheumatic heart disease through prompt recognition, treatment of GAS pharyngitis, supporting access to clean water for adequate hygiene and access to healthcare [26, 27].

### Strength and limitations of the study

Our study recruited children from semi-urban and rural areas representing a middle-class and a lower socioeconomic class populations, thus representing the population of the majority of people in Dar Es Salaam. Recall bias might have affected the recollection of URTIs in the previous six months thus potentially affecting its impact on the subclinical RHD. Furthermore, we were not able to ascertain any history of treatment with antibiotics (or other potential confounders) which may have confounded the association between individual characteristics and the detection of subclinical RHD.

## Conclusion

Subclinical RHD detected by echocardiography is common in primary school children in Tanzania. Mitral valve pathology was the only type of valvular lesion found. Factors associated with subclinical RHD were age greater than 9 years, recurrent URTI, and poor hygiene. These characteristics may be considered as possible criteria for identifying children at highest risk for RHD and thus likely to benefit from screening and secondary prevention programs. Prospective studies are needed to understand the natural history of RHD detected by echocardiography in Sub-Saharan Africa and to understand how screening programs can be utilized by government health programs to reduce the burden of RHD.

## Data Availability

Data and materials are available upon request to the authors.

## Abbreviations

AMVL: Anterior mitral valve leaflet
BMI: Body mass index
DRC: Democratic Republic of Congo
GAS: Group A streptococcus
MR: Mitral regurgitation
RF: Rheumatic fever
URTI: Upper respiratory tract infection
